# Predictive value of body composition and core symptoms in schizophrenia for cardiorespiratory fitness: CORTEX-SP study

**DOI:** 10.1192/j.eurpsy.2022.514

**Published:** 2022-09-01

**Authors:** M. Tous-Espelosin, N. Iriarte Yoller, C. Pavón, P.M. Sanchez, A. Sampedro, S. Maldonado-Martín

**Affiliations:** 1 Faculty of Education and Sport-Physical Activity and Sport Sciences Section. University of the Basque Country (UPV/EHU), Department Of Physical Education And Sport, Vitoria-Gasteiz, Spain; 2 Osakidetza, Alava´s Psychiatric Hospital, Mental Health Network, Vitoria-Gasteiz, Spain; 3 Faculty of Psychology and Education, University of Deusto, Department Of Methods And Experimental Psychology, Bilbao, Spain

**Keywords:** cardiorespiratory fitness, body composition, core symptoms, schizophrénia

## Abstract

**Introduction:**

Cardiorespiratory fitness (CRF) can be directly measured and assessed by the cardiopulmonary exercise test (CPET) or estimated from different field tests as the Modified Shuttle Walking Test (MSWT). The CRF in schizophrenia (SP) population may be altered due to sex, age, body composition and core symptoms variables. However, the extent to which each domain influences CRF in this pathology is still unknown.

**Objectives:**

To analyze the predictive value of body composition and core symptoms in SP for CRF.

**Methods:**

Participants (N = 144, 41.7 ± 10.3 yr old) with SP were assessed with (1) body mass index and fat percentage; (2) upright bicycle ergometer using an incremental ramp protocol and the MSWT; and (3) positive and negative symptoms of the disease [“Positive and Negative Syndrome Scale” (PANSS) and “The Brief Negative Symptom Scale” (BNSS)]. In the Stepwise Multiple Regression analyses, those variables which correlated (Spearman’s Rho) significantly with each CFR scores were included

**Results:**

Lower negative symptoms (*P<*0.001) and positive PANSS (*P*=0.035) predicted V̇O_2peak_ (L·min^−1^) (R^2^=28.3%). Lower negative symptoms (*P<*0.001), positive PANSS (*P*=0.006) and fat body mass (*P<*0.001) explained V̇O_2peak_ (mL·kg^−1^·min^−1^) (R^2^=46.5%). MSWT was predicted (R^2^=58.9%) by lower negative symptoms (*P*=0.001), body mass (*P<*0.001) and total PANSS (*P*=0.004).

**Conclusions:**

In patients with SP significantly higher CRF was detected in those with lower negative and positive symptoms, as well as lower body mass. Exercise interventions for improving CRF should be promoting in this population for a better control of core symptoms.

**Disclosure:**

No significant relationships.

